# Evidence of Genomic Diversification in a Natural Symbiotic Population Within Its Host

**DOI:** 10.3389/fmicb.2022.854355

**Published:** 2022-03-01

**Authors:** Clotilde Bongrand, Eric Koch, Daniel Mende, Anna Romano, Susannah Lawhorn, Margaret McFall-Ngai, Edward F. DeLong, Edward G. Ruby

**Affiliations:** ^1^Kewalo Marine Laboratory, SOEST, University of Hawai‘i at Mānoa, Honolulu, HI, United States; ^2^Department of Oceanography, SOEST, University of Hawai‘i at Mānoa, Honolulu, HI, United States

**Keywords:** *Vibrio fischeri*, comparative genomic, *Euprymna scolopes*, symbiosis, population biology

## Abstract

Planktonic cells of the luminous marine bacterium *Vibrio fischeri* establish themselves in the light-emitting organ of each generation of newly hatched *Euprymna scolopes* bobtail squid. A symbiont population is maintained within the 6 separated crypts of the organ for the ∼9-month life of the host. In the wild, the initial colonization step is typically accomplished by a handful of planktonic *V. fischeri* cells, leading to a species-specific, but often multi-strain, symbiont population. Within a few hours, the inoculating cells proliferate within the organ’s individual crypts, after which there is evidently no supernumerary colonization. Nevertheless, every day at dawn, the majority of the symbionts is expelled, and the regrowth of the remaining ∼5% of cells provides a daily opportunity for the population to evolve and diverge, thereby increasing its genomic diversity. To begin to understand the extent of this diversification, we characterized the light-organ population of an adult animal. First, we used 16S sequencing to determine that species in the *V. fischeri* clade were essentially the only ones detectable within a field-caught *E. scolopes*. Efforts to colonize the host with a minor species that appeared to be identified, *V. litoralis*, revealed that, although some cells could be imaged within the organ, they were <0.1% of the typical *V. fischeri* population, and did not persist. Next, we determined the genome sequences of seventy-two isolates from one side of the organ. While all these isolates were associated with one of three clusters of *V. fischeri* strains, there was considerable genomic diversity within this natural symbiotic population. Comparative analyses revealed a significant difference in both the number and the presence/absence of genes within each cluster; in contrast, there was little accumulation of single-nucleotide polymorphisms. These data suggest that, in nature, the light organ is colonized by a small number of *V. fischeri* strains that can undergo significant genetic diversification, including by horizontal-gene transfer, over the course of ∼1500 generations of growth in the organ. When the resulting population of symbionts is expelled into seawater, its genomic mix provides the genetic basis for selection during the subsequent environmental dispersal, and transmission to the next host.

## Introduction

*Vibrio (Aliivibrio) fischeri* is a marine gram-negative bacterium that can establish a symbiosis within the light-emitting organ of the Hawaiian bobtail squid *Euprymna scolopes*, providing bioluminescence to its partner each night in exchange for nutrients. When the aposymbiotic (i.e., symbiont-free) juvenile squid hatches from its egg, it specifically harvests *V. fischeri* cells from the ambient seawater (Nawroth et al., 2017; Visick et al., 2021). These bacteria enter and migrate through different tissue environments to finally reach and colonize the epithelium-lined crypts of the host’s nascent light organ. Each lobe of the bilobed organ bears three pores, each leading to a separate crypt (McFall-Ngai, 2014), increasing the chance for a multi-strain inoculation, and reducing the opportunity for strain-strain competition. Every day at dawn, ∼95% of the symbiont population is expelled, and the remaining ∼5% regrow, repopulating the crypts within hours (Lee and Ruby, 1994). Therefore, the diversity of strains present in the light organ can arise from (i) the diversity of inoculating *V. fischeri* strains present in the local environment (Wollenberg and Ruby, 2009), (ii) the stochasticity of the initial colonization events (Bongrand and Ruby, 2018), and (iii) the subsequent evolution of those strains as they go through many daily cycles of population depletion and regrowth within the crypts. The genomic divergence resulting from this latter dynamic makes it of interest to determine the number of genomically distinct strains present in the light organ of a fully grown (i.e., >3 month-old) squid.

The presence of multiple, co-occurring strains of a symbiont species within a host, as well as the nature of their strain-level genetic differences, have become of increasing interest as a means to better understand the dynamics of host health and metabolism (Ansorge et al., 2019; Hinzke et al., 2021). The monospecificity of the *E. scolopes/V. fischeri* system simplifies the investigation of genomic differences between co-occurring strains in a natural association (Bongrand et al., 2016, 2020), as well as of the potential for behavioral interactions between these strains (Speare et al., 2018). In particular, because the differences between genomes are small due to the short periods of diversification, they may be difficult to determine using a purely metagenomic approach.

Previously, we have shown that the genome synteny of *V*. *fischeri* is surprisingly well conserved among *E*. *scolopes* light-organ symbionts collected across different geographic areas, and over a span of more than a decade (Bongrand et al., 2016). In addition, two colonization behaviors have been described during experimental inoculations of juvenile squid: (i) a dominant (D-type) behavior, in which one strain eclipses another during a co-infection of the host, and (ii) a sharing (S-type) behavior, in which both strains persist after a co-inoculation (Bongrand et al., 2016, 2020). One characteristic of D-type behavior is that such strains appear to reach and colonize the light-organ crypts more quickly than S-type strains (Bongrand and Ruby, 2018). Interestingly, both D-type and S-type strains can be found in field-caught animals (Wollenberg and Ruby, 2009), an unexpected outcome that may result from a sequential exposure of the newly hatched host to different *V. fischeri* strains in the environment (Bongrand and Ruby, 2018). Taken together, these characteristics of the squid-vibrio system make it a good model with which to study both colonization dynamics, and intra-species diversification within the symbiotic population of a host (Yawata et al., 2014; Duar et al., 2017; McLean et al., 2018; Bongrand and Ruby, 2019).

A previous rep-PCR study estimated that at least 6 to 8 strains were present in the light organ of a field-caught adult squid (Wollenberg and Ruby, 2009). However, under laboratory inoculation procedures, a single light organ can reportedly be colonized by as many as a hundred distinct mutant derivatives of the same strain (Brooks et al., 2014), indicating the potential for multi-strain populations in the symbiosis. In our study, we used culture-dependent and independent approaches to confirm that *V. fischeri* is essentially the only species detectable in the light organ of *E. scolopes*. In addition, we estimated the number of co-occurring strains of this species by whole-genome sequencing, using Illumina platform MiSeq (2*300bp), of around 80 isolates, obtained as colony-forming units (CFU) from one lobe (i.e., 3 crypts) of the light organ of a field-caught adult squid, collected off the island of Oahu, Hawaii, United States. All of the individual strains analyzed were identified as *V. fischeri*, and were closely associated with one of three genomic clusters of strains. While there was a substantial level of diversity in the total number and presence/absence of individual genes between and among these clusters, we found little evidence of significant evolutionary drift, as indicated by single nucleotide polymorphisms (SNP).

## Results and Discussion

### Analysis of the Specificity of Light-Organ Occupancy

**FIGURE 1:**
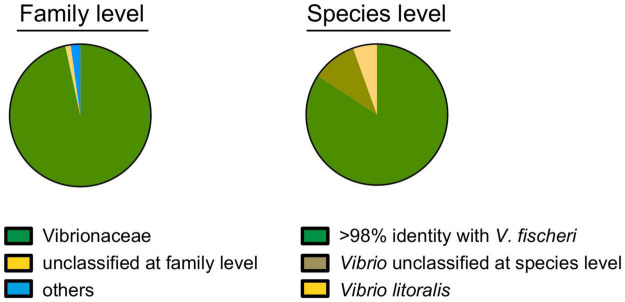
Identification of the bacterial population within a symbiotic light organ at the family **(left)** and species **(right)** levels, based on the sequence of the V3-V4 regions of the 16S ribosomal RNA gene. Of the 176,118 total reads, 173,915 (99%) and 152,745 (87%) could be assigned to a family or species, respectively.

*Vibrio fischeri* has been described as the only bacterial species present in the light organ of the squid *E. scolopes*, based on the phenotypic identification of hundreds of CFU isolated from dozens of animals (Boettcher and Ruby, 1990; Ruby and Lee, 1998). The only reported exception to this specificity is that some isolates of the closely related (i.e., 97% 16S sequence identity) species *Vibrio logei* (Bang et al., 1978), subsequently proposed for renaming (Ast et al., 2009), can stably colonize *E. scolopes* juveniles, and are found co-occurring with *V. fischeri* as light-organ symbionts in other species of sepiolid squids (Fidopiastis et al., 1998). Here, a metagenomic approach using an Illumina platform MiSeq (2*300bp) intended to identify the symbionts present in the light organs of field-caught specimens of *E. scolopes*. Specifically, we sequenced the V3-V4 region of the 16S RNA genes of members of the bacterial population present in one of the two symbiont-containing core tissues, representing half of the bilaterally symmetrical light organ. Two adult squids from Maunalua Bay, Oahu, HI were sampled; one was collected in 2005 (Wollenberg and Ruby, 2009) and the other was obtained in 2019. Analyses of both produced the same outcome. Limiting the analysis to bacterial families identified in more than 1000 of the 176,118 total reads (i.e., >0.5%), only two categories were detected: “*Vibrionaceae*” (99%) and “unclassified at a family level” (1%) (Figure 1). *V. fischeri* strain ES114 (Boettcher and Ruby, 1990) has been used as a genome reference for the species because of its complete, closed (Ruby et al., 2005), and subsequently updated (Mandel et al., 2008) sequence. This strain encodes twelve copies of the 16S DNA, comprising two distinguishable sequences that are 98% identical. In the available genomes of another 42 *V. fischeri* strains, including those draft genomes providing only one 16S sequence, there were a total of 4 versions of the typically analyzed hypervariable V3-V4 region (Wang and Qian, 2009), all of which share >98% identity. Therefore, we considered that, conservatively, strains with 98% or more identity with these *V. fischeri* sequences over this region belonged to this species. This assumption is consistent with the sequence similarity threshold for species differentiation described in the literature (Kim et al., 2014).

Within this context, 79%, 13%, and 5% of the 16S rRNA sequencing reads corresponded to, respectively: (i) *V. fischeri*, (ii) “unclassified at species level” and (iii) *V. litoralis* (Figure 1). *V. litoralis* is currently represented by two draft genome sequences in the database. The V3-V4 region for these two are identical to each other, and 96% identical to *V. fischeri*. Only two isolates of *V. litoralis* have been described (Nam et al., 2007), and essentially nothing else has been published about this species. It is difficult to distinguish with certainty two species with V3-V4 region sequences with >96% identity (Mysara et al., 2017), especially if the read-quality for accurate taxonomy assignment is taken into account (Bokulich et al., 2013). It has also been reported that there can be a limitation in the use of 16S-gene sequence comparisons for species-level taxonomy; for example, sequences belonging to the same species can be as little as 94% identical, while sequences from different species can be up to 97% identical (Lan et al., 2016). Thus, it is not unlikely that the 5% of 16S reads, indicated as *V. litoralis* (Figure 1), recovered from the light organ might, in fact, represent strains of *V. fischeri*. This result is consistent with an absence of *V. litoralis* among the 80 symbiont CFU sequenced in this study (see below), although this species is easily cultured (Nam et al., 2007). In addition, *V. litoralis* does not encode bacterial luciferase, and produces no luminescence (data not shown), and luminescence has been shown to be a strict requirement for persistence of *V. fischeri* in the light organ (Koch et al., 2014). Taken together, these data support the view that it is unlikely that members of this species are a resident population in the light organ. Overall these results confirm the specificity of the *E. scolopes/V. fischeri* association, consistent with the reported selective pressure in this niche for the symbiont (Nyholm et al., 2000).

**FIGURE 2:**
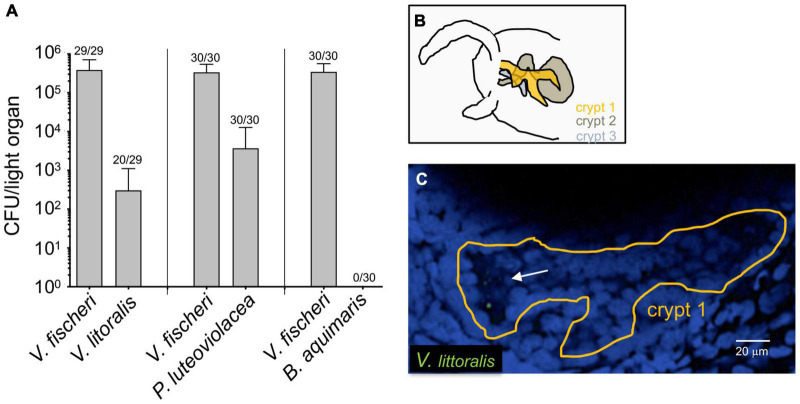
Colonization of newly hatched *E. scolopes* by environmental bacteria. Juvenile squid were inoculated with pairs of strains consisting of *V. fischeri* and either *V. litoralis* (*n* = 29), *P. luteoviolacea* (*n* = 30) or *B. aquimaris* (*n* = 30) in three replicates (*n* = total number of animals in each condition). **(A)** The average number of colony-forming units (CFU) present in co-colonized light organs after 24 h. The number above each bar indicates the proportion of animals in which each strain was found; except for the *B. aquimaris* experiment, in which all the squids were only colonized with ES114; only animals in which both strains were detected were used to calculate the average. **(B)** Schematic of the interior of one side of a juvenile light organ, indicating the position of the three crypts. **(C)** One slice of a confocal microscope image of a crypt (tissue nuclei stained blue with TOPRO) in which 8-10 GFP-labeled *V. litoralis* cells (green) can be seen (arrow). A z-stack movie of the entire crypt can be found in Supplementary Video 1.

To determine whether other bacterial species can enter the light organ transiently, especially when the juvenile is being initially colonized, we inoculated aposymbiotic squids with *V. fischeri* and one of three environmental marine isolates (Figure 2A). Because of the possibility that this species might be present in an adult light organ (Figure 1), we chose a strain of *V. litoralis*, as well as another Gram-negative bacterium that associates with marine larvae, *Pseudoalteromonas luteoviolacea*, and a Gram-positive marine bacterium *Bacillus aquimaris* (Figure 2A). Twenty-four hours after exposure to the inoculum, each animal was rinsed and homogenized, and the homogenate plated for CFU. While *B. aquimaris* was not detected, CFU of the other species were often found; however, in those squids inoculated with these species, there were only 0.01–0.5% as many CFU as in a colonization by *V. fischeri*. Nyholm et al. described a 2-h “permissive window” post initial exposure, during which < 2 μm-diameter beads or bacteria that were not *V. fischeri* could be found present in the newly hatched juvenile’s light organ (Nyholm et al., 2002). Here we describe the presence of such non-symbiotic bacteria in the crypts after 24 h of initial exposure. Both results suggest that other bacterial species, while present for a short period of time during the juvenile stage, don’t go on to colonize the squids; i.e., they are not capable of symbiosis, or of competing with *V. fischeri*. Our results further suggest that such non-symbiotic strains can remain longer than a few hours in the light organ. To determine where *V. litoralis* cells were within the light organ, we GFP-labeled a strain of this species and visualized co-inoculated squid by confocal microscopy (Figures 2B,C), and confirmed that a few dozen GFP-labeled *V. litoralis* cells were dispersed sporadically within the crypts of the light organ (Supplementary Video 1). To ask whether the presence of the natural symbiont had an impact on colonization by these strains, we performed both single- and co-inoculations of juvenile squid with *V. fischeri* and one of the three other strains, and analyzed their success after 24 and 48 h (Supplementary Figure 1). Generally, the quantity of each strain, with or without the presence of *V. fischeri*, was similar, suggesting there was no evidence of either a strong competition or complementation by the presence of the natural symbiont on these environmental strains. However, a few squid harbored a very low number of *B. aquimaris* when *V. fischeri* was absent, compared to none when *V. fischeri* was present. This finding may suggest that newly hatched squid are more permissive to other bacteria when they don’t encounter their symbiont. Indeed, the symbiont aggregates and concentrates at the pores of the light organ, giving itself an advantage compare to other species present in the environment (Nyholm et al., 2000), being absent will give a stochastic opportunity for other bacteria to reach and enter the pores of the light organ. In addition, we realize the inoculum used in these assays are also much higher than the one found in the environment which can increase the number of CFU present in the light organ for the non-symbiotic strains. We also wondered whether the non-*V. fischeri* species persisted in the light organ of the squid, and found that there were fewer of these bacteria at 48 h than at 24 h (Supplementary Figure 1B), suggesting that while they can enter, non-symbiotic environmental strains are just transient in the light organ of the squid. Nevertheless, they may indicate that other species can transiently provide new genetic material for the *V. fischeri* population that can take up and incorporate exogenous DNA by natural transformation (Pollack-Berti et al., 2010).

Overall, one aim in this study was to determine whether there is evidence for a significant number of an unrecognized species in the squid light organ, one that had been overlooked because it was not culturable by the methods used for isolating *V. fischeri*. However, no species other than *V. fischeri* appeared to be persistently present among the symbiont population.

### Genomic Diversification Within the Symbiont Population

In nature, a juvenile *E. scolopes* typically becomes colonized by only a few cells of *V. fischeri* (Wollenberg and Ruby, 2012), drawn from an environmental pool of genomicly distinct strains (Bongrand et al., 2016). For the subsequent ∼9 months that the host lives, the symbiont population undergoes a daily cycle of expulsion and proliferate each morning (Schwartzman and Ruby, 2016). Thus, we predicted that an analysis of the population of a field-caught adult host light organ would reveal: (i) how many strains had initiated the population, (ii) what was their relative success, and (iii) whether there was evidence that the progeny had diverged from their ancestral genome. While studies selecting for better colonization have been performed in experimentally colonized animals, to date they have been initiated by inoculation with a single strain (Schuster et al., 2010; Soto and Nishiguchi, 2014; Pankey et al., 2017). Our interest was in examining the outcome of a multiple-strain colonization; however, arranging such a colonization experimentally can be challenging, even with as few as three strains (Bongrand and Ruby, 2018). Therefore, we decided to examine a snapshot in time of a naturally colonized adult light organ (i.e., >3 months after colonization) by genome-sequencing 80 CFU obtained from one side of the bilobed light organ of a 23-mm mantle-length, field-caught female (Wollenberg and Ruby, 2009). Of the initial 80 sequenced strains, 8 yielded DNA sequences that were of poor quality, so we excluded them from further analysis.

**FIGURE 3:**
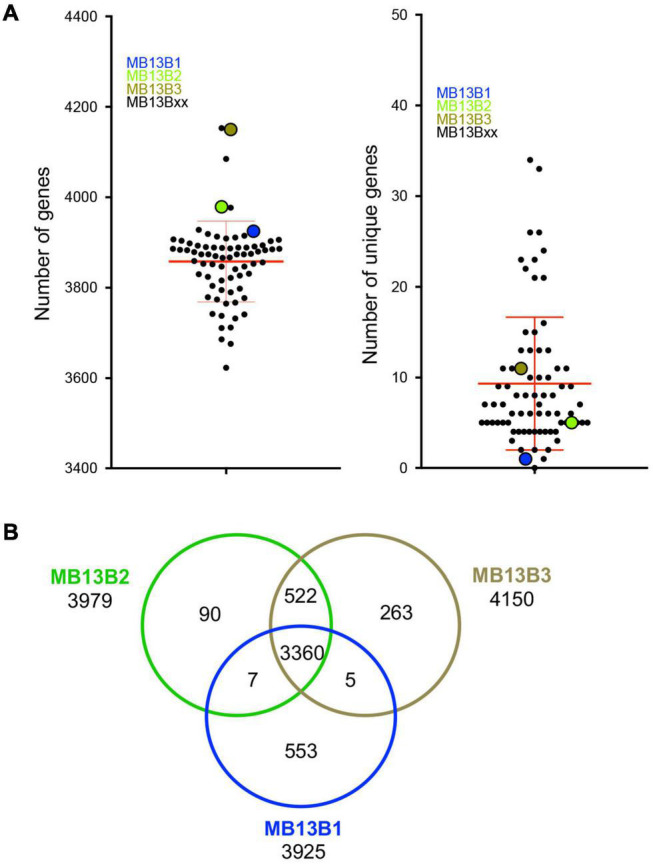
**(A)** Total number of genes (left) and number of unique genes (right) for each MB13B isolate compared to all other 74 isolates. **(B)** Venn diagram indicating the number of total, shared and unique genes in a comparison between strains MB13B1, MB13B2 and MB13B3.

The number of predicted genes in each isolate’s genome spanned from 3623 to 4153, with a mean of 3858 (Figure 3A), which is within the published range for *V. fischeri* (Bongrand et al., 2016). The number of unique genes in a given genome was calculated to be between 0 and 34 among the 72 isolates; however, because the data are from draft genomes, the actual number of unique genes may be different. Thus, the relatively low numbers of unique encoded proteins may be due to (i) limitations in the process of sequence assembly, and/or (ii) horizontal gene transfer (HGT) events experienced by members of the population, as well as gene loss amongst these strains during symbiosis. Indeed, the possibility of extensive HGT in the dense symbiont population is supported by the facts that (i) *V. fischeri* is naturally competent under conditions found in the symbiosis (Pollack-Berti et al., 2010) and (ii) HGT, such as plasmid transfer, has been reported between strains in the host (Dunn et al., 2005). The three previously characterized strains (Wollenberg and Ruby, 2009) isolated from this same light-organ lobe – MB13B1, MB13B2 and MB13B3 – encode 3915, 3979 and 4150 proteins, including 553, 90 and 263 unique proteins, respectively, when compared to each other (Figure 3B); strains MB13B2 and MB13B3 are more closely related to each other than either is to MB13B1 (Bongrand et al., 2016). Interestingly, the number of unique proteins decreased to 34 when the additional 72 available sequenced *V. fischeri* genomes were considered, a result that is consistent with a small number of ancestral/parent strains in the inoculum.

**FIGURE 4:**
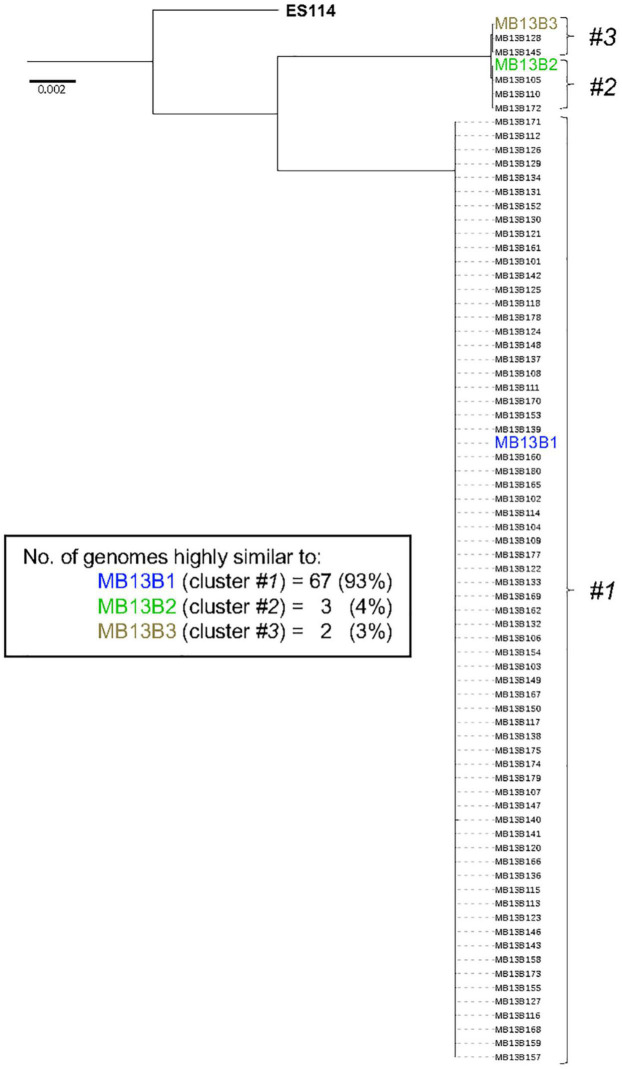
Phylogenetic tree based on the core genome sequence of 72 *V. fischeri* isolates from one lobe of an adult *E. scolopes* light organ. The tree is rooted using *V. fischeri* strain ES114; the locations of the 3 previously sequenced strains (colored) are indicated.

The core genome of *V. fischeri* consists of 2308 genes and, on a phylogenetic tree based on these core genes, all the sequenced isolates grouped with one of the three previously sequenced strains (Figure 4). This finding is confirmed by a pairwise gene-distance analysis of the genes belonging to the core genome. Interestingly only 30 of the 2308 core genes showed a pairwise distance between 0.1 and 0.5, suggesting that there was a low rate of evolutionary divergence in the light organ over the several months that the symbiosis had replicated in the crypts (estimated at approximately 500 generations). Because the intrinsic mutation rate of *V. fischeri* is reported to be low (Dillon et al., 2017), we predicted that there would be a low level of divergence among the progeny of the initial colonizers of this portion of the light-organ, unless these symbionts also experienced a significant degree of selection pressure.

The groups represented by strains MB13B1, MB13B2, and MB13B3 account for 93%, 4%, and 3% (respectively) of the total isolates sequenced, suggesting that strain MB13B1 is more highly represented in this half of the organ. One possible explanation for this disproportional representation is the available space and, thus, potential for growth, a strain has when proliferating within a crypt (Essock-Burns et al., 2020). A recent study showed that in a polyclonal population present in the tubeworm symbiosis, the relative proportion of strains was more a result of their growth in the host (Polzin et al., 2019), a hypothesis that may hold here as well. Such a scenario would also help explain strain MB13B1’s numerical advantage when strains MB13B2 and MB13B3 have been described as more effective at initiating a colonization (Bongrand and Ruby, 2018). This proportion could also be unintentionally biased during the picking of the colonies from the plate. A more accurate identification of the proportion of each strain may well be obtained by using a metagenomic approach, rather than one based on CFU selection.

**FIGURE 5:**
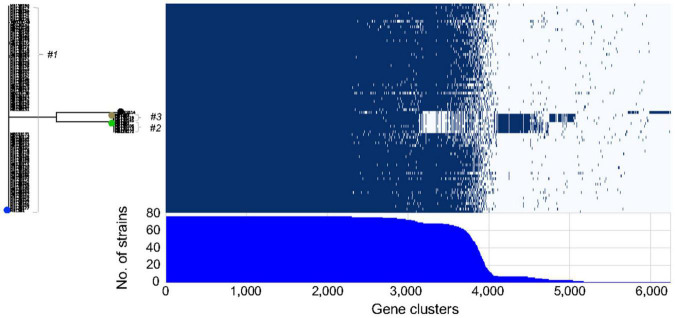
Roary matrix indicating the presence or absence of genes in 6246 clusters among 75 light-organ isolates from lobe MB13B within clusters *#1-3*. Strain ES114, isolated from another light organ, is included for comparison (black dot). The positions of previously sequenced strains are indicated by blue (MB13B1), green (MB13B2), and brown (MB13B3) dots.

Finally, an examination of the presence and absence of genes among the different isolates (Figure 5) suggested that considerable divergence within the initial inoculating strains had occurred post-colonization. The core genome among the 72 sequenced isolates consists of 2308 genes, which is low when compared to the 3170 genes found for 14 strains that were significantly more biologically and ecologically diverse (Bongrand et al., 2016). This result will be an underestimate since some genes will be considered missing because of the technology used (short read) and the quality of the assembly. However, this could also result from frequent HGT occurring between the bacteria residing, or transiently occurring, in the light organ, and is consistent with the reported large number of gene loss and gain events that *Vibrio* spp. typically have during their evolution (Lin et al., 2018). Taken together, the data here support the conclusion that 3 strains initially colonized one lobe of squid MB13, and that their progeny have evolved into pseudo-clones harboring highly related, yet unique, genes sets. In a study of *V. cholerae* strains, the population in an individual patient was reported to experience more HGT than point mutations, which led the authors to conclude that strain divergence is due primarily to gene exchange within the *V. cholerae* population (Levade et al., 2017). The work presented here suggests that *V. cholerae* and *V. fischeri* may evolve in a similar manner within their respective hosts; however, while the cholera infection occupies the gut (a single communicating lumen) and is often clonal (Levade et al., 2017) the *V. fischeri* light-organ population typically derives from a few strains, likely corresponding to the several distinct crypt spaces it occupies (McFall-Ngai, 2014; Essock-Burns et al., 2020). It has also been shown in the gut microbiota that strains recovered from the same individual were more similar to each other than to strains from other individuals, and that the diversity within an individual host came principally from changes in gene content rather than from SNPs (Vatanen et al., 2018).

In our study, we confirmed that one side of the squid’s bilobed light organ can become colonized by three strains. Because the frequency of co-colonization of a crypt by two different strains can be as low as ∼1% (Bongrand and Ruby, 2018), it seems likely that, in the ocean, the light organs of newly hatched squid are generally colonized by around 6 strains (Wollenberg and Ruby, 2009). Over the subsequent several months, the progeny of each strain within a crypt can diverge into many pseudo-clones characterized by a number of unique genes, but a low level of SNPs. Overall this genomic analysis suggests that *V. fischeri* may begin to go through an elimination of non-functional sequences after associating with its host (Bobay and Ochman, 2017).

Overall, this study aimed to begin to understand the mechanisms underlying the population diversity that can result from the association between a host and its horizontally transmitted symbiont. We used an amplicon-sequencing approach to confirm the single-species specificity of this symbiosis, and then used an Illumina platform MiSeq (2*300bp) to sequence cultured isolates to infer the genomic diversity of this population. Using these conclusions as a foundation, future studies will first examine additional symbiont populations in multiple adult light organs to establish hypotheses that describe the dynamics of population diversity in nature. Second, analyses of both the genomes of bacterial isolates and population metagenomes should determine the proportion of the different strains present in the light organs. Finally, studies should use the non-intrusive sampling afforded by the daily expulsion event to track the nature of the symbiont population of individual light organs over time, to determine whether there are age-specific dynamics of population diversification in a natural symbiotic relationship.

## Materials and Methods

### Bacterial Strains

We performed this study on bacteria isolated from the crypt-containing central-core tissue of a single light-organ lobe of an adult specimen of *E. scolopes* (MB13) caught in Maunalua Bay, Oahu, HI and frozen in glycerol in 2005 (Wollenberg and Ruby, 2009). The internal tissue sample was dissected out, rinsed with sterile seawater, and frozen at −80°C. This sample was chosen because draft genomes were already available for three strains (MB13B1, MB13B2, and MB13B3) isolated from it, and included two phenotypically distinct groups (Bongrand et al., 2016). Three isolates of other species of marine bacteria, identified as *Vibrio litoralis* strain DSM17657 (Nam et al., 2007), *Pseudoalteromonas luteoviolacea* strain HI1 (Huang et al., 2012) and *Bacillus aquimaris* strain TF-12 (Yoon et al., 2003) were also used in the study. Colonies of the latter two species could be easily distinguished from the *Vibrio* species by their purple and yellow-orange pigmentation, respectively.

### 16S RNA Gene Analyses

The symbiont-containing light-organ lobe tissue designated MB13B (Wollenberg and Ruby, 2009) was shipped to Omega Bioservices (Norcross, GA, United States) in ethanol, and processed to identify 16S gene sequences (Bioproject ID: PRJNA796326; BioSample accession number SAMN24847873). DNA was extracted using the Mag-Bind Universal Pathogen DNA kit (Omega), the library prepared with the KAPA HIFI PCR for 16S kit (KAPABIOSYSTEMS), and the 16S V3-V4 regions were sequenced using the Illumina primers (IlluminaF: CCTACGGGNGGCWGCAG; IlluminaR: GACTACHVGGGTATCTAATCC) on a Illumina MiSeq. The coverage per sample was ∼50K reads, with a paired-end read format of 2*300. Omega Bioservices also performed sequence classification using the Illumina’s BaseSpace 16S rRNA application module, Illumina-curated version (May 2013) using the Greengenes taxonomic database in parallel with the Ribosomal Database Project (RDP) (Wang et al., 2007). The accuracy for species classification was estimated to be 98%.

### Next Generation Sequencing

An aliquot of the −80°C frozen glycerol stock containing an homogenate of the right-side central core of the light organ of squid MB13 was spread onto Luria-Bertani Salt (LBS) agar medium containing (per liter) 20 g NaCl, 50 ml of 1 M Tris–HCl (pH 7.5), 10 g Bacto-Tryptone, 5 g yeast extract, and 12 g agar, and left overnight at 28°C. The following day, 80 colonies were picked at random, and cultured in LBS broth. After shaking overnight at 28°C, 500 μL of culture (OD_600nm_ between 1 and 2) was centrifuged, and the pellet resuspended in 400 μL lysis buffer containing 40 mM EDTA, 50 mM Tris–HCl (pH 8.3) and 0.75 M sucrose, and frozen at –80°C. The suspension was thawed, and incubated with 1 mg lysozyme (Sigma Aldrich, St. Louis, MO, United States) per ml for 30 min at 37°C. Then, proteinase K (Roche, Basel, Switzerland) and SDS were added to 0.8 mg/ml and 1% final concentrations, respectively, and incubated for 2 h at 55°C. The released DNA was purified with a chemagic Magnetic Separation Module I (Perkin Elmer, Waltham, MA, United States), and quantified with the Quant-iT Picogreen dsDAN kit (Invitrogen). These genomic DNA samples were normalized, and libraries were prepared with the Nextera XT 96 DNA Library preparation kit (Illumina, San Diego, CA, United States). The sequencing was performed with a MiSeq V3 (Illumina) for 600 cycles, providing 22–25 million paired-end reads (2*300 bp) per run (sequence information can be found at Bioproject ID: PRJNA796703; BioSample accession numbers can be found in Supplementary Table 1).

### Bioinformatic Analysis of the Genomic DNA

Genome assemblies were performed with SPAdes (Nurk et al., 2013), and annotated with prokka (Seemann, 2014). CheckM (Parks et al., 2015) was used to evaluate the completeness and potential contamination of the genomes, and genomes below 90% completeness were excluded. All genomes showed contamination estimates below 5%. High quality assemblies as well as publicly available genomes of *V. fischeri* strains MB13B1, MB13B2, MB13B3, and ES114 were used as input for a Roary pangenome analysis (Page et al., 2015). Roary was run using default parameters. FastTree2 (Price et al., 2010) was used to calculate a core genome tree using the concatenated alignment provided by Roary.

### Colonization of the Squid Light Organ

The GFP-encoding plasmid pVSV102 (Dunn et al., 2006) was inserted into *V. litoralis* strain DSM17657 by conjugation as previously described (Stabb and Ruby, 2002). Overnight cultures of *V. litoralis* and *V. fischeri* were started in LBS medium, supplemented with kanamycin (50 μg per ml) when necessary to maintain pVSV102. Otherwise, bacteria were grown in seawater tryptone (SWT) media (Boettcher and Ruby, 1990). Bacteria were grown with shaking at 28°C until mid-exponential phase (∼0.5 OD_600nm_), and diluted to a targeted concentration of 5000 cells per ml in 50 ml of filter-sterilized ocean water (FSOW) into which newly hatched juvenile squids were transferred. Squid were inoculated at room temperature with either one or both bacterial species, and maintained for 24 or 48 h. When the experiment lasted 48 h, the water was changed with fresh FSOW after 24 h. At the end of the experiment, the squid were rinsed individually twice for 1 min, and once for 5 min, in vials containing 4 ml FSOW, before being either frozen at –80°C, or anesthetized in 2% ethanol and fixed in 4% paraformaldehyde in mPBS (50 mM sodium phosphate buffer with 0.45 M NaCl, pH 7.4). The frozen animals were homogenized and aliquots plated on SWT agar plates, and the number of CFU that arose was used to calculate the symbiont population size in the light organ. Colonies of the GFP-labeled *V. litoralis* were differentiated from *V. fischeri* under a fluorescence dissecting scope, and colonies of *P. luteoviolacea* and *B. aquimaris* were identified by their distinct pigmentation. The fixed animals were rinsed four times for 30 min each in mPBS, and dissected. They were counterstained with TOPRO-3 (1:1000) in 1% TRITON-X100 mPBS, then rinsed four times for 15 min in mPBS. Light organs were mounted on a slide using Vectashield mounting medium, and imaged on a Zeiss LSM710 laser-scanning confocal microscope.

## Data Availability Statement

The datasets presented in this study can be found in online repositories. The names of the repository/repositories and accession number(s) can be found below: https://www.ncbi.nlm.nih.gov/, SAMN24847873; https://www.ncbi.nlm.nih.gov/, SAMN24907221–SAMN24907300.

## Ethics Statement

The University of Hawaii Institutional and Animal Care and Use Committee (IACUC) is only allowed to review research using vertebrate animals. The communicating author has a letter from the University veterinarian that states the use of cephalopods in the research conducted in this study would pass IACUC standards if they were allowed to formally review it.

## Author Contributions

CB, EK, ER, and MM-N contributed to conception and design of the study. DM performed the bioinformatic analysis. CB and SL performed the animal colonizations. AR and CB performed the experimental sequencing. AR and ED provided training and facilities. CB wrote the first draft of the manuscript. CB, DM, and ER wrote sections of the manuscript. All authors contributed to manuscript revision, read, and approved the submitted version.

## Conflict of Interest

The authors declare that the research was conducted in the absence of any commercial or financial relationships that could be construed as a potential conflict of interest. The reviewer ES declared a past co-authorship with one of the authors ER, to the handling editor.

## Publisher’s Note

All claims expressed in this article are solely those of the authors and do not necessarily represent those of their affiliated organizations, or those of the publisher, the editors and the reviewers. Any product that may be evaluated in this article, or claim that may be made by its manufacturer, is not guaranteed or endorsed by the publisher.

## Supplementary Material

Supplementary Video 1 Colonization of newly hatched *E. scolopes* by *V. fischeri* and *V. litoralis* (GFP-labeled). A z-stack of the left side of a juvenile light organ (tissue nuclei stained blue with TOPRO) in which many GFP-labeled *V. litoralis* cells can be seen. No such fluorescent structures were observed in videos of light organs that were either uncolonized or exposed to bacteria without GFP labels.
