# Supplementary material for: Evidence of Genomic Diversification in a Natural Symbiotic Population Within Its Host
Source: Front Microbiol. 2022 Mar 1;13:854355. doi: 10.3389/fmicb.2022.854355 (PMC8922018; doi:10.3389/fmicb.2022.854355)
Supplement: Supplementary file 1 [file Table_1.pdf]

Supplementary Table S1. References for the *V. fischeri* strains isolated from the glycerol stock MB13B, containing an homogenate of the right-side central core of the light organ of squid MB13.

Strain designation	Bioproject ID	Accession number
MB13B101	PRJNA796703	SAMN24907221
MB13B102	PRJNA796703	SAMN24907222
MB13B103	PRJNA796703	SAMN24907223
MB13B104	PRJNA796703	SAMN24907224
MB13B105	PRJNA796703	SAMN24907225
MB13B106	PRJNA796703	SAMN24907226
MB13B107	PRJNA796703	SAMN24907227
MB13B108	PRJNA796703	SAMN24907228
MB13B109	PRJNA796703	SAMN24907229
MB13B110	PRJNA796703	SAMN24907230
MB13B111	PRJNA796703	SAMN24907231
MB13B112	PRJNA796703	SAMN24907232
MB13B113	PRJNA796703	SAMN24907233
MB13B114	PRJNA796703	SAMN24907234
MB13B115	PRJNA796703	SAMN24907235
MB13B116	PRJNA796703	SAMN24907236
MB13B117	PRJNA796703	SAMN24907237
MB13B118	PRJNA796703	SAMN24907238
MB13B119	PRJNA796703	SAMN24907239
MB13B120	PRJNA796703	SAMN24907240
MB13B121	PRJNA796703	SAMN24907241
MB13B122	PRJNA796703	SAMN24907242
MB13B123	PRJNA796703	SAMN24907243
MB13B124	PRJNA796703	SAMN24907244
MB13B125	PRJNA796703	SAMN24907245
MB13B126	PRJNA796703	SAMN24907246
MB13B127	PRJNA796703	SAMN24907247
MB13B128	PRJNA796703	SAMN24907248
MB13B129	PRJNA796703	SAMN24907249
MB13B130	PRJNA796703	SAMN24907250
MB13B131	PRJNA796703	SAMN24907251
MB13B132	PRJNA796703	SAMN24907252
MB13B133	PRJNA796703	SAMN24907253
MB13B134	PRJNA796703	SAMN24907254
MB13B135	PRJNA796703	SAMN24907255
MB13B136	PRJNA796703	SAMN24907256
MB13B137	PRJNA796703	SAMN24907257
MB13B138	PRJNA796703	SAMN24907258
MB13B139	PRJNA796703	SAMN24907259
MB13B140	PRJNA796703	SAMN24907260
MB13B141	PRJNA796703	SAMN24907261
MB13B142	PRJNA796703	SAMN24907262
MB13B143	PRJNA796703	SAMN24907263

MB13B144	PRJNA796703	SAMN24907264
MB13B145	PRJNA796703	SAMN24907265
MB13B146	PRJNA796703	SAMN24907266
MB13B147	PRJNA796703	SAMN24907267
MB13B148	PRJNA796703	SAMN24907268
MB13B149	PRJNA796703	SAMN24907269
MB13B150	PRJNA796703	SAMN24907270
MB13B151	PRJNA796703	SAMN24907271
MB13B152	PRJNA796703	SAMN24907272
MB13B153	PRJNA796703	SAMN24907273
MB13B154	PRJNA796703	SAMN24907274
MB13B155	PRJNA796703	SAMN24907275
MB13B156	PRJNA796703	SAMN24907276
MB13B157	PRJNA796703	SAMN24907277
MB13B158	PRJNA796703	SAMN24907278
MB13B159	PRJNA796703	SAMN24907279
MB13B160	PRJNA796703	SAMN24907280
MB13B161	PRJNA796703	SAMN24907281
MB13B162	PRJNA796703	SAMN24907282
MB13B163	PRJNA796703	SAMN24907283
MB13B164	PRJNA796703	SAMN24907284
MB13B165	PRJNA796703	SAMN24907285
MB13B166	PRJNA796703	SAMN24907286
MB13B167	PRJNA796703	SAMN24907287
MB13B168	PRJNA796703	SAMN24907288
MB13B169	PRJNA796703	SAMN24907289
MB13B170	PRJNA796703	SAMN24907290
MB13B171	PRJNA796703	SAMN24907291
MB13B172	PRJNA796703	SAMN24907292
MB13B173	PRJNA796703	SAMN24907293
MB13B174	PRJNA796703	SAMN24907294
MB13B175	PRJNA796703	SAMN24907295
MB13B176	PRJNA796703	SAMN24907296
MB13B177	PRJNA796703	SAMN24907297
MB13B178	PRJNA796703	SAMN24907298
MB13B179	PRJNA796703	SAMN24907299
MB13B180	PRJNA796703	SAMN24907300
