# Supplementary figures and images for: Evidence of Genomic Diversification in a Natural Symbiotic Population Within Its Host
Source: Front Microbiol. 2022 Mar 1;13:854355. doi: 10.3389/fmicb.2022.854355 (PMC8922018; doi:10.3389/fmicb.2022.854355)

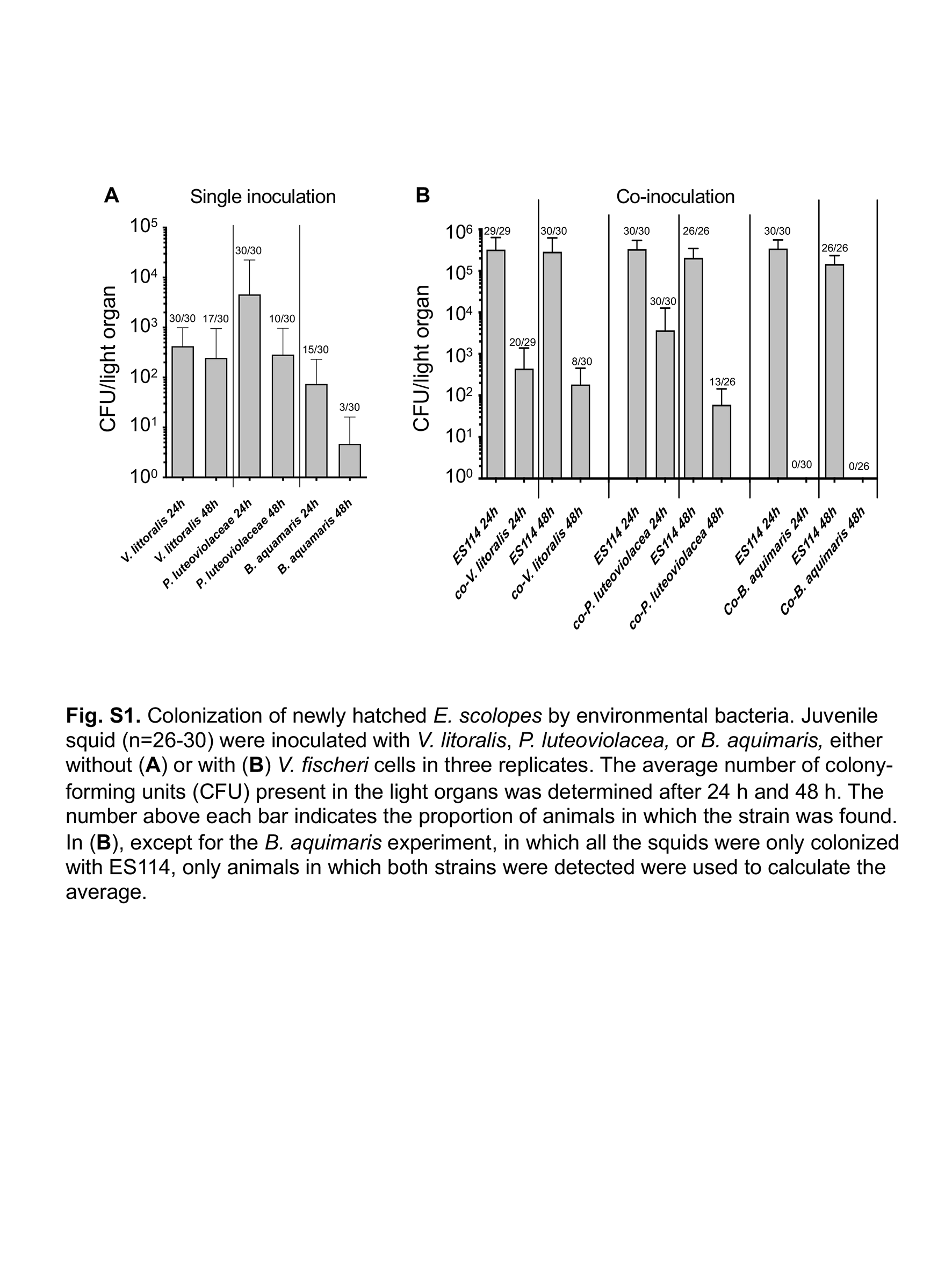

Supplement: Supplementary file 2 [file Image_1.tif]
